# Compound Heterozygosity for a Novel Frameshift Variant Causing Fatal Infantile Liver Failure and Genotype–Phenotype Correlation of *POLG* c.3286C>T Variant

**DOI:** 10.3390/ijns7010009

**Published:** 2021-02-05

**Authors:** Kanokwan Sriwattanapong, Kitiwan Rojnueangnit, Thanakorn Theerapanon, Chalurmpon Srichomthong, Thantrira Porntaveetus, Vorasuk Shotelersuk

**Affiliations:** 1 Genomics and Precision Dentistry Research Unit, Department of Physiology, Faculty of Dentistry, Chulalongkorn University, Bangkok 10330, Thailand; kanokwan.sr@chula.ac.th (K.S.); theerapanon.t@gmail.com (T.T.); 2 Department of Pediatrics, Faculty of Medicine, Thammasat University, Pathumthani 12120, Thailand; rkitiwan@tu.ac.th; 3 Center of Excellence for Medical Genomics, Medical Genomics Cluster, Department of Pediatrics, Faculty of Medicine, Chulalongkorn University, Bangkok 10330, Thailand; chalurmpon_s@hotmail.com (C.S.); vorasuk.s@chula.ac.th (V.S.); 4 Excellence Center for Genomics and Precision Medicine, King Chulalongkorn Memorial Hospital, The Thai Red Cross Society, Bangkok 10330, Thailand

**Keywords:** failure to thrive, hepatopathy, liver, mitochondria, polymerase, metabolic disorder, newborn

## Abstract

A variant in the *POLG* gene is the leading cause of a heterogeneous group of mitochondrial disorders. No definitive treatment is currently available. Prenatal and newborn screening have the potential to improve clinical outcome of patients affected with *POLG*-related disorders. We reported a 4-month-old infant who presented with developmental delay, fever, and diarrhea. Within two weeks after hospital admission, the patient developed hepatic failure and died. Liver necropsy demonstrated an extensive loss of hepatocytes and bile duct proliferations. Trio-whole exome sequencing identified that the patient was compound heterozygous for a novel frameshift variant c.3102delG (p.Lys1035Serfs*59) and a common variant c.3286C>T (p.Arg1096Cys) in *POLG* (NM_002693.3) inherited from the mother and father, respectively. The c.3102delG (p.Lys1035Serfs*59) was a null variant and classified as pathogenic according to the American College of Medical Genetics and Genomics Standards and Guidelines. Prenatal genetic screenings using rapid whole exome sequencing successfully detected the heterozygous c.3286C>T variant in the following pregnancy and the normal alleles in the other one. Both children had been healthy. We reviewed all 34 cases identified with the *POLG* c.3286C>T variant and found that all 15 compound heterozygous cases had two missense variants except our patient who had the truncating variant and showed the earliest disease onset, rapid deterioration, and the youngest death. All homozygous cases had disease onset before age 2 and developed seizure. Here, we report a novel *POLG* variant expanding the genotypic spectrum, demonstrate the successful use of exome sequencing for prenatal and neonatal screenings of *POLG*-related disorders, and show the genotype–phenotype correlation of the common c.3286C>T variant.

## 1. Introduction

Mitochondria are double membrane bound organelles that play important roles for energy production and cell survival. The *POLG* gene (OMIM *174763) encodes the catalytic subunit of DNA polymerase gamma (pol γ), which is essential for mitochondrial DNA replication, repair, and maintenance [1,2]. Alterations in the *POLG* gene are the most common cause of inherited mitochondrial disorders, with up to 2% of the population having the variants [2,3]. Six major *POLG*-related disorders, showing overlapping phenotypes and a wide range of disease severity and onset from infancy to adulthood, comprise: (1) the childhood myocerebrohepatopathy spectrum (MCHS) presenting from the first few months of life to the age of three years with developmental delay, lactic acidosis, myopathy, and failure to thrive; (2) Alpers–Huttenlocher syndrome (AHS) characterized by childhood onset of severe encephalopathy, epilepsy, and hepatic failure; (3) myoclonic epilepsy myopathy sensory ataxia (MEMSA) presenting with epilepsy, myopathy, and ataxia without ophthalmoplegia, and including spinocerebellar ataxia with epilepsy (SCAE); (4) the ataxia neuropathy spectrum (ANS), presenting with ataxia and neuropathy, and including mitochondrial recessive ataxia syndrome (MIRAS) and sensory ataxia neuropathy dysarthria and ophthalmoplegia (SANDO); (5) autosomal recessive progressive external ophthalmoplegia (arPEO), characterized by progressive weakness of the extraocular eye muscle; and (6) autosomal dominant progressive external ophthalmoplegia (adPEO), which is an adult onset of external eye muscle weakness.

Genetic testing is important for diagnosing the *POLG*-related disorders [4]. Treatment is currently limited to symptom management and supportive care. Prenatal and neonatal screening and early intervention for the *POLG*-related disorders can ameliorate symptoms, reduce irreversible damage, and improve the clinical outcomes of the patients. If left undiagnosed, the newborns may suffer severe consequences including developmental delay, organ damage, and disability. Here, we reported a patient who was compound heterozygote for a novel frameshift c.3102delG (p.Lys1035Serfs*59) variant and a previously reported c.3286C>T (p.Arg1096Cys) variant in *POLG* (NM_002693.3). The patient had a severe childhood myocerebrohepatopathy spectrum and died at 4 months old. For the family, prenatal screenings of the two following pregnancies were successfully performed by rapid whole exome sequencing. Here, we also reviewed the phenotype and genotype of an additional 33 patients reported to have the c.3286C>T variant.

## 2. Case Report

The proband was the second child of a non-consanguineous couple. He was born at 39-week gestation by normal delivery. His birth weight was 3196 g (25–50th centile) and Apgar scores were 9 and 10 at 5 and 10 min, respectively. During the first three months, he was in good health. At 4 months old, the patient started showing symptoms. He had fever, vomiting, diarrhea, jaundice and pale stool. He had a delay in development. He was unable to roll over, grasp, or lift his head. His weight was 6.2 kg (50th centile), height 64 cm (50–75th centile), and head circumference 39 cm (50th centile). Marked icteric sclera and liver enlargement (liver span 10 cm) were observed. A liver function test showed cholestatic jaundice with acute hepatic failure. Total bilirubin was 145.35 µmol/L (reference < 21), direct bilirubin 109.44 µmol/L (<3.4), lactate 4 mmol/L (<2), AST 257 U/L (5–55), ALT 139 U/L (5–45), ALP 139 U/L (150–420), albumin 2.3 g/dL (3.5–5.5), globulin 1.5 g/dL (2.3–3.5), prothrombin time 48.7 s (11.5–15.3), partial thromboplastin time 76.2 s (35.1–46.3 s), and international normalized ratio (INR) 4.24 s (0.86–1.22). The CBC, BUN, creatinine, ammonia, electrolyte, and blood sugar were within normal ranges. Liver ultrasound revealed hepatosplenomegaly, diffuse increase in parenchymal echotexture, diffuse thickening of gallbladder wall, and bile sludge. Diisopropyl iminodiacetic acid (DISIDA) scan showed poor hepatic intake with no evidence of gall bladder and bowel activities. Comprehensive metabolic tests including fatty acid oxidation defect, tyrosinemia type I deficiency, and citrin deficiency were negative. Tandem mass spectrometry detected increased levels of methionine, phenylalanine, and tyrosine. Viral antibody tests of hepatitis A, B and C, herpes simplex, and Epstein–Barr viruses and PCR of adenovirus and enterovirus were negative. The PCR for cytomegalovirus was positive. 

He developed severe ascites, fulminant hepatic failure, hepatic encephalopathy, and severe coagulopathy. Although the Single Pass Albumin Dialysis (SPAD) was administered, his condition progressively worsened. The patient deceased 2 weeks after hospital admission. Liver necropsy showed an extensive loss of hepatocytes, bile duct proliferation, and a large number of foamy cells. Viral cytopathic change was not observed. Whole exome sequencing (WES) was performed as described in previous studies [5,6]. Genomic DNA was extracted from peripheral blood leukocytes. WES was performed using an Illumina Hiseq4000 sequencer at Macrogen Inc. (Seoul, Korea). The variants were filtered following these criteria: (1) passed all quality filters during the variant-calling process, (2) had a read depth > 10, (3) located in the coding regions and canonical splice sites of genes related to the hepatic failure according to the Human Phenotype Ontology, HP:0001399 [7], and (4) had <1% minor allele frequency in the Genome Aggregation Database (gnomAD), Exome Variant Server, 1000 Genomes Project Consortium, dbSNPs, and in-house database of 2166 Thai exomes. Only the variants in *POLG* passed the filtering criteria. The variant was called novel if it was not listed in the Human Gene Mutation Database [8]. Pathogenicity of the filtered variant was classified according to the American College of Medical Genetics and Genomics (ACMG) standards and guidelines [9]. The pathogenic variant was validated by Sanger sequencing using primers listed in Table S1.

**Figure 1:**
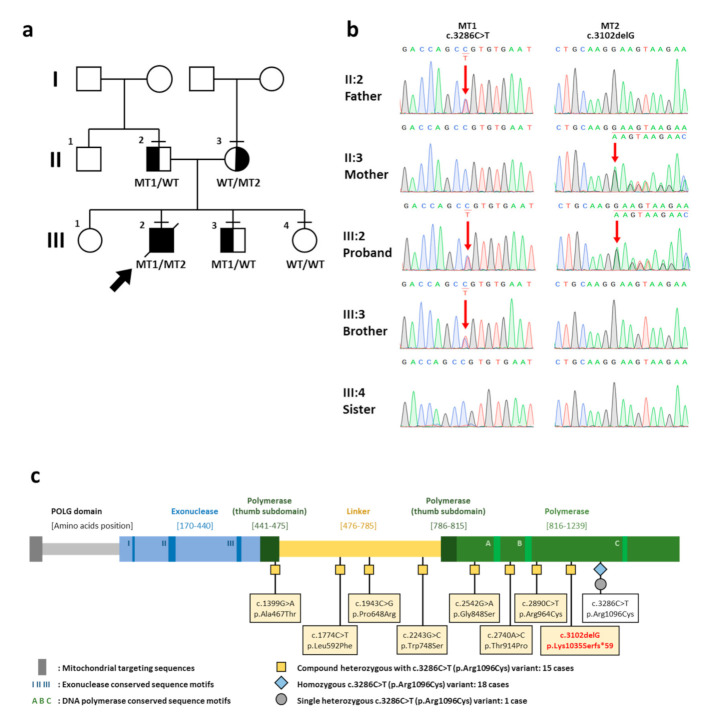
Family pedigree, chromatograms, and schematic diagram of the POLG protein. (**a**) Family pedigree. The proband is indicated by an arrow. A horizontal line above each symbol indicates the family members participated in genetic testing. (**b**) Chromatograms show that the proband possesses the compound heterozygous variants, c.3286C>T and c.3102delG, in *POLG* (NM_002693.3). The heterozygous variant, c3286C>T, is observed in the father and the proband’s younger brother. The heterozygous variant, c.3102delG, in *POLG* is present in the mother. Red arrows indicate the *POLG* variants. The younger sister possesses two normal alleles. (**c**) Schematic diagram of the POLG protein. The *POLG* variants reported to be compound heterozygous with the c.3286C>T (p.Arg1096Cys) variant are tagged with yellow square. The homozygous and single heterozygous c.3286C>T (p.Arg1096Cys) variants are tagged with blue diamond and grey circle, respectively. The novel c.3102delG (p.Lys1035Serfs*59) variant identified in the proband is shown in red letters. Functional domain is demonstrated above the diagram. I, II, and III indicate the conserved sequence motifs for 3′–5′ exonuclease function. A, B, and C indicate the conserved sequence motifs for DNA polymerase function. WT, wild type allele; MT1, c.3286C>T allele; MT2, c.3102delG allele.

Trio-WES identified that the patient harbored the compound heterozygous variants in the *POLG* gene. A novel frameshift variant, c.3102delG (p.Lys1035Serfs*59), in exon 19 of *POLG* (NM_002693.3, ClinVar SCV001245410) was detected on the maternal allele. The c.3102delG (p.Lys1035Serfs*59) was a null variant and classified as pathogenic (PVS1, PM2, PM3) according to the ACMG Standards and Guidelines [9]. On the paternal allele, the missense *POLG* variant, c.3286C>T (p.Arg1096Cys), in exon 21 was identified (ClinVar SCV001245409; dbSNP rs201732356). This variant was previously reported in several studies (Table 1). Both the c.3102delG and c.3286C>T variants are located in the polymerase domain of the POLG protein (Figure 1).

Prenatal testing for the third and fourth pregnancies was performed by rapid WES (Illumina’s NextSeq^TM^ 500, Excellence Center of Medical Genomics, Bangkok, Thailand) using amniocyte DNA at 16-week gestation. The third fetus was found to be heterozygous for the missense *POLG* variant, c.3286C>T (p.Arg1096Cys), while the fourth fetus was found with two wild-type alleles. The duration from amniocentesis to molecular diagnosis of these two pregnancies was less than seven days. Sanger sequencing of blood derived DNA was performed to confirm WES results. At present, both children are healthy.

Thirty-four patients, including our proband, had been identified with the c.3286C>T variant in *POLG* (Table 1). Of those, 18 were homozygous, 15 were compound heterozygous, and one was single heterozygous. Among 15 compound heterozygous cases for the c.3286C>T variant, 14 cases had the missense variant on another allele while the proband was the only one with the truncating variant. He had the most severe phenotype, earliest onset, and youngest death, compared with the other compound heterozygous patients. In addition, we observed that all homozygous patients with the c.3286C>T variant had disease onset before the age of 2 years and developed seizure.

## 3. Discussion

We reported an infant affected with severe MCHS. The patient was in good health at birth. The onset of illness was at the age of 4 months. Due to hepatic failure, the patient died at 4 months old or 2 weeks after his first hospital admission. Seizure was not present. Due to rapid disease progression, it is possible that the patient might not survive long enough to develop seizure [2].

Trio WES identified that the proband was compound heterozygous for a novel c.3102delG (p.Lys1035Serfs*59) and a known c.3286C>T (p.Arg1096Cys) variant in *POLG* (NM_002693.3) which was inherited from the mother and father, respectively. Several studies have tried to establish the genotype–phenotype correlation of *POLG-*related disorders, but none were successful. The same variant can lead to a wide range of different symptoms. *POLG* truncating variants including nonsense and frameshift variants have been reported to cause a spectrum of phenotypes in many studies [2,25,26]. The *POLG* Pathogenicity Prediction Server (http://polg.bmb.msu.edu) has been established to predict the pathogenicity of variants based on five distinct clusters. However, the variants affecting different functional domains or clusters of POLG can have either exacerbating or compensatory effects. These complicate the prediction of disease phenotype or prognosis on the basis of observed variants [27]. In this study, we decided to target our review into the previously reported *POLG* c.3286C>T variant. Thirty-four patients (including the proband) with the c.3286C>T variant were identified. Of those, 18 were homozygous, 15 were compound heterozygous, and one was single heterozygous [1,3,4,10,11,12,13,14,15,16,17,18,19,20,21,22,23,24]. A single heterozygous c.3286C>T variant was reported in a patient affected with progressive external ophthalmoplegia without neurological symptoms [24]. Among 15 compound heterozygous cases for the c.3286C>T variant, our proband had the earliest disease onset and death (4 months old). Consistent with the phenotype, the proband was the only one who was compound heterozygous for the truncating variant while the other 14 cases were compound heterozygous for the missense variant. This suggests that the truncating variant when found with the missense c.3286C>T variant can cause early onset and rapid progression of disease. We also observed that all homozygous cases for the c.3286C>T variant showed symptoms before the age of 2 years and developed seizure. In contrast, a majority of the compound heterozygous cases had late clinical presentation until adulthood and seizure was not common.

*POLG* is essential for mitochondrial DNA to replicate and repair. Alterations in *POLG* cause mitochondrial DNA depletion or deletion in several organs leading to diverse clinical manifestations. However, the same variant can lead to mitochondrial DNA deletion, depletion or both, complicating phenotype–genotype correlation. In addition, other factors including genetic modifiers, immunity, and environmental effects such as mitochondrial toxins and infections can modify the *POLG* disease phenotype [28,29]. A previous study demonstrated that the homozygous c.3286C>T variant in *POLG* caused a depletion of mitochondrial DNA and a reduction in cytochrome c oxidase activity [11]. A functional study in a patient with Alpers syndrome and compound heterozygosity for a nonsense p.Glu873* and a missense p.Ala467Thr variant showed that more than 95% of functional *POLG* mRNA was derived from the missense allele and less than 5% of mRNA from the nonsense allele. The nonsense variant was found to be subjected to a nonsense-mediated decay and the Alpers phenotype was a consequence of a functional missense allele and selectively nonfunctional nonsense allele [27,30]. Based on the above evidence, it is speculated that the more severe phenotype in our proband, compared with the other compound heterozygous cases, could be a deleterious consequence of the truncating variant combined with the milder effect of the missense variant. The variants were expected to reduce, and interfere with, protein function, resulting in organ malfunction.

At present, no evidence-based therapies are available for *POLG*-related disorders [2]. Genetic testing is essential for prenatal and neonatal screening. Here, the compound heterozygous *POLG* variants were successfully detected by trio WES and the two following prenatal diagnoses were achieved by rapid WES. These prove the efficacy of WES for prenatal screening of *POLG*-related disorders. Newborn screening enables a presymptomatic diagnosis. This could lead to dose reduction or avoidance of medications metabolized by hepatic enzymes such as valproic acid and sodium valproate. These medications can precipitate or accelerate liver disease. In addition, similar to some other mitochondrial diseases, physical factors such as fever and dehydration may lead to a sudden deterioration and should be avoided.

In conclusion, we report the novel frameshift *POLG* variant expanding the genotypic spectrum of *POLG*-related disorders and demonstrate the association between the truncating variant and disease severity. We also show that prenatal exome sequencing is effectively used for screening *POLG*-related disorders. 

## Figures and Tables

**Table 1:** The patients reported with the variant, c.3286C>T (p.Arg1096Cys), in exon 21 of the *POLG* gene (NM_002693.3).

No.	Age of Onset	Age at Report	Age at Death	Nationality	Genotype				Clinical Manifestations			
					The Other Variant	Expected Amino Acid Change of the Other Variant	Exon	Domain	Liver Failure	Seizure	Other Signs and Symptoms	Reference
**Homozygous variant**												
1.	birth	4 y	NA	United Arab Emirates	NA	NA	NA	NA	-	+	subtle neurodevelopmental problem, develop left-sided weakness	Mohamed et al., 2011 [10]
2.	6 w	4 m	11 m	United Arab Emirates	NA	NA	NA	NA	NA	+	Alpers syndrome, hepatomegaly, abnormal liver function, mid hypotonia	Mohamed et al., 2011 [10]
3.	4 m	6 m	NA	United Arab Emirates	NA	NA	NA	NA	NA	+	Alpers syndrome, severe hypotonia, modulate liver involvement	Mohamed et al., 2011 [10]
4.	4 m	6 m	NA	United Arab Emirates	NA	NA	NA	NA	+	+	Alpers syndrome, severe neurological disease, chronically elevated liver enzymes	Mohamed et al., 2011 [10]
5.	5 m	5 m	NA	United Arab Emirates	NA	NA	NA	NA	-	+	hypotonia, lethargy, moderate developmental delay	Mohamed et al., 2011 [10]
6.	5 m	11 m	NA	United Arab Emirates	NA	NA	NA	NA	+	+	-	Mohamed et al., 2011 [10]
7.	5 m	5 m	15 m	NA	NA	NA	NA	NA	+	+	Alpers syndrome, non-specific encephalopathy and blindness, severe coagulopathy, hypoglycemia, epilepsy, hepatopathy	Ashley et al., 2008 [11]
8.	8 m	8 m	18 m	Afghanistan	NA	NA	NA	NA	+	+	altered sensorium, hypotonia, mild hepatomegaly	Bijarnia-Mahay et al., 2014 [12]
9.	9 m	9 m	NA	Arab states	NA	NA	NA	NA	NA	+	dementia, encephalopathy	Tang et al., 2011 [4]
10.	<12 m	3 y	NA	United Arab Emirates	NA	NA	NA	NA	-	+	hypotonia, refractory epilepsia partialis continua, severe neurological disability	Mohamed et al., 2011 [10]
11.	12 m	12 m	NA	Arab states	NA	NA	NA	NA	+	+	-	Tang et al., 2011 [4]
12.	12 m	12 m	NA	NA	NA ^a^	NA ^a^	NA	NA	NA	+	Alpers syndrome, dementia	Wong et al., 2008 [1]
13.	NA	12 m	NA	Arab states	NA	NA	NA	NA	NA	+	Alpers syndrome, multifocal therapy-refractory epilepsy, hippocampal sclerosis, COX-negative fibers, reduced mtDNA copy number, mtDNA deletions.	Stewart et al., 2010 [13]
14.	13 m	13 m	NA	NA	NA	NA	NA	NA	NA	NA	Choreoathetosis; myoclonus (epileptic and non-epileptic); intermittent, myoclonic jerks sometimes in sleep, worsened by illness; abnormal neurotransmitters	Papandreou et al., 2018 [14]
15.	14 m	14 m	NA	Saudi arabia	NA	NA	NA	NA	+	+	Alpers syndrome, epilepsia partialis continua (EPC), hypotonia	Kentab, 2019 [15]
16.	24 m	24 m	NA	Arab states	NA	NA	NA	NA	NA	+	developmental delay, elevated transaminases, lactic acidosis	Tang et al., 2011 [4]
17.	24 m	9 y	NA	NA	NA ^a^	NA ^a^	NA	NA	+	NA	encephalopathy, myoclonus achalasia	Horvath et al., 2006 [16]
18.	NA	NA	NA	European	NA	NA	NA	NA	NA	+	Leigh syndrome	De Kovel et al., 2016 [17]
**Compound Heterozygous Variant with Frameshift Variant**												
19.	4 m	4 m	4 m	Thai	c.3102delG	p.Lys1035Serfs*59	19	P	+	-	developmental delay, hepatic failure	This study
20.	5 m	5 m	11 m	NA	c.2542G>A	p.Gly848Ser	16	P	+	NA	encephalopathy, hypotonia	Stumpf et al., 2013 [3]
21.	<12 m	<12 m	14 m	NA	c.2740A>C	p.Thr914Pro	18	P	+	NA	Alpers syndrome, hypotonia, myoclonic epilepsy, respiratory insufficiency, hepatopathy, choreoathetosis, ataxia, ataxic nystagmus	Ashley et al., 2008 [11]
22.	24 m	24 m	NA	Arab states	c.2542G>A	p.Gly848Ser	16	P	+	NA	developmental delay, hypotonia, dementia/encephalopathy, exercise intolerance, muscle weakness, easy fatigability, ptosis, gastrointestinal reflux, delayed gastric emptying, cyclic vomiting, elevated transaminases, lactic acidosis, short statue, failure to thrive	Tang et al., 2011 [4]
23.	17 y	42 y	NA	England	c.1399G>A	p.Ala467Thr	7	L	NA	NA	chronic progressive external ophthalmoplegia, ptosis, peripheral neuropathy, sensory and motor neuronopathy, distal and proximal neurogenic change	Lax et al., 2012 [18]
24.	25 y	49 y	NA	England	c.2243G>C	p.Trp748Ser	13	L	NA	NA	chronic progressive external ophthalmoplegia, ptosis, peripheral neuropathy, epilepsy, severe sensory and moderate motor neuronopathy	Lax et al., 2012 [18]
25.	26 y	45 y	NA	European	c.1399G>A	p.Ala467Thr	7	L	NA	+	myoclonic seizures, epilepsy	Whittaker et al., 2015 [19]
26.	26 y	55 y	NA	European	c.2243G>C	p.Trp748Ser	13	L	NA	+	myoclonic and tonic-clonic seizures, epilepsy, multifocal epileptiform abnormalities	Whittaker et al., 2015 [19]
27.	40 y	61 y	NA	NA	c.2890C>T	p.Arg964Cys	18	P	NA	NA	chronic progressive external ophthalmoplegia, ptosis	Heighton et al., 2019 [20]
28.	42 y	42 y	NA	NA	c.1399G>A	p.Ala467Thr	7	L	NA	NA	severe ptosis, myopathy, cerebella dysfunction, peripheral neuropathy	Yu-Wai-Man et al., 2013 [21]
29.	48 y	48 y	NA	NA	c.1774C>T	p.Leu592Phe^b^	10	L	NA	NA	sensory ataxic neuropathy, dysarthria, ophthalmoplegia, dysphagia	Kurt et al., 2012 [22]
30.	49 y	55 y	NA	NA	c.1943C>G	p.Pro648Arg	10	L	NA	NA	sensory ataxic neuropathy, dysarthria, ophthalmoparesis	Masingue et al., 2019 [23]
31.	53 y	55 y	NA	NA	c.1943C>G	p.Pro648Arg	10	L	NA	NA	progressive external ophthalmoplegia, myopathy	Horvath et al., 2006 [16]
32.	54 y	54 y	NA	NA	c.2243G>C	p.Trp748Ser	13	L	NA	NA	ataxia, epilepsy, peripheral neuropathy, cognitive impairment	Yu-Wai-Man et al., 2013 [21]
33.	NA	NA	NA	NA	c.2243G>C	p.Trp748Ser	13	L	NA	NA	mitochondrial recessive ataxia syndrome	Masingue et al., 2019 [23]
**Single heterozygous variant**												
34.	23 y	23 y	NA	Italian	NA	NA	NA	NA	NA	NA	sporadic progressive external ophthalmoplegia	Agostino et al., 2003 [24]

P, polymerase domain; L, linker domain; w, week; m, month; y, year; NA, not available or not applicable; +, present; -, not present. ^a^ Presenting with the homozygous variant, c.3708G>T (p.Gln1236His), in *POLG.* ^b^ p.Leu592Phe was reported as p.Leu591Phe by Kurt et al., 2012 [22].

## Data Availability

The data presented in this study are available in the supplementary material.

## Supplementary Materials

The following are available online at https://www.mdpi.com/2409-515X/7/1/9/s1, Table S1. Primer sequences used for confirmation of variants, c.3102delG and c.3286C>T, identified through WES.
