# Supplementary material for: Compound Heterozygosity for a Novel Frameshift Variant Causing Fatal Infantile Liver Failure and Genotype–Phenotype Correlation of POLG c.3286C>T Variant
Source: Int J Neonatal Screen. 2021 Feb 5;7(1):9. doi: 10.3390/ijns7010009 (PMC7930966; doi:10.3390/ijns7010009)
Supplement: Supplementary file 1 [file IJNS-07-00009-s001.pdf]

Supplementary Material

Compound Heterozygosity for a Novel Frameshift Variant Causing Fatal Infantile Liver Failure and Genotype–Phenotype Correlation of *POLG* c.3286C>T Variant

Kanokwan Sriwattanapong [1], Kitiwan Rojnueangnit [2], Thanakorn Theerapanon [1], Chalurmpon Srichomthong [3,4], Thantrira Porntaveetus [1,*] and Vorasuk Shotelersuk [3,4]

Table S1. Primer sequences used for confirmation of variants, c.3102delG and c.3286C>T, identified through WES.

Primer Name	Mutation		Primer Sequence (5'to 3')	Product Size (bp)
Maternal *POLG*	c.3102delG	Forward	AGA CGT AGC AAT GCT CTC AAG	381
		Reverse	CCC GTG GAA TGA TGG TGA TAA	
Paternal *POLG*	c.3286C>T	Forward	GGT CAA GAG GTT GGT GAT CTG	329
		Reverse	CCT GTG GAC CTT ACC AAT GTT	
